# Families’ Experiences of Surrogate Decision-Making and Challenges of Shared Decision-Making: A Cross-Sectional Study

**DOI:** 10.7759/cureus.79866

**Published:** 2025-03-01

**Authors:** Kanako Yamamoto

**Affiliations:** 1 Critical Care Nursing, St. Luke's International University, Tokyo, JPN

**Keywords:** decision aid, end-of-life, family, intensive care unit, palliative care, patient, shared decision-making, surrogate, surrogate decision-making

## Abstract

Introduction: Family members who make surrogate decisions tend to be emotionally burdened and may have difficulty making decisions that fit the patient’s wishes. It is unclear what support family members making surrogate decisions expect from health professionals.

This study aims to clarify the information needed for surrogate decision-making and the support needs of healthcare professionals.

Methods: This cross-sectional study used questionnaires. Family members with experience in making surrogate decisions for treatments that affected the patient's life and prognosis were included. Participants were recruited using an online panel through a private research company in Japan. For the survey items, 60 participants were asked to specify one case in which they made a surrogate decision and describe the resources they used to support their decision-making. In addition, participants were assessed on a 10-point scale to determine their sense of satisfaction with the decision, whether the decision was per the patient’s wishes, and their level of emotional burden. Knowledge and understanding of life-sustaining treatments were also investigated.

Results: Family members tended to make decisions that were more aligned with the patient's desires than with theirs (P = 0.005). In addition, decisions that prioritized the patient's desires tended to result in higher levels of satisfaction after the decision was made (r = 0.349, P < 0.01). Family members who understood the treatment being given to the patient tended to experience lower psychological stress after the decision was made (r = -0.394, P < 0.01).

The information that families considered important for making decisions on the patient’s behalf included whether the patient would regain consciousness (70%) and the patient's age (66.7%).

Conclusion: The findings suggest that medical professionals should encourage family members to make decisions in keeping with the patient's wishes and provide the patient's information, including risk information, to help them understand the treatment. In addition, considering the mental stress and burden on family members, it is necessary to consider the process of discussion among family members while providing mental support to facilitate the decision-making process.

## Introduction

When an adult patient with a loss or decline in decision-making capacity needs to make treatment or care decisions, surrogates make decisions on their behalf. In general, the surrogate decision-maker is a trusted person designated by the patient or the patient's family [1,2]. Concerns have been raised about the accuracy of decision-making by a surrogate [3]. For example, the surrogate’s wishes may take precedence over the patient’s wishes [4,5]. In addition, surrogate decision-makers are burdened by the extent of their responsibility [6] and are more likely to have depression and post-traumatic stress disorder when making treatment decisions that affect the patient’s survival or prognosis [2].

The medical-clinical terms healthcare professionals use make it difficult for surrogate decision-makers to understand treatment [7]. Approximately 50-70% of surrogate decision-makers have a poor understanding of the treatment provided to the patient [8]. Surrogate decision-makers may choose costly and futile treatments by failing to consider the patient’s values and wishes, thereby increasing the burden on the patient [9]. However, if there is an advance care planning (ACP) process in which patients disclose their treatment wishes and goals and share them with healthcare providers and surrogates, they report significantly less conflict regarding surrogate decision-making [10,11]. Having prior ACP discussions with a family member or healthcare professional can help respect patient autonomy and reduce the emotional burden on surrogates. There is no clearly defined and widely recognized definition of surrogate decision-making quality. Assessing the quality of surrogate decision-making is difficult because there are many differences depending on whom the decision is judged to benefit, that is, the patient, the surrogate, or both. There is also a lack of clarity regarding the quality of surrogate decision-making. However, I believe that it is important to support both the patient’s right to independence and the surrogate decision-maker in reducing conflicts regarding decision-making. To achieve this, the preparation and decision-making processes must be enhanced [1].

So far, we know that surrogate decision-making does not increase satisfaction by simply encouraging patients to understand the treatment. Factors related to the quality of surrogate decision-making include clarifying patient values and identifying preferences [12]. Healthcare professionals play an important role in helping surrogates make decisions in line with the patient’s wishes. However, many healthcare professionals struggle to support surrogate decision-makers. Lack of support and communication also creates conflict between healthcare professionals and surrogate decision-makers [13]. The support needs of surrogate decision-makers must be clarified, and healthcare professionals must understand how to support surrogate decisions. This will help identify ways to provide educational support for both. Few previous studies have detailed the decision-making process of surrogates. In particular, the kind of support families require from healthcare professionals for surrogate decision-making is unclear. By conducting a survey of families who have actually made surrogate decisions, it may be possible to identify the gap between the support provided by medical staff and that provided by family members. The provision of optimal care to families is of paramount importance, as it has the potential to mitigate the substantial stress associated with surrogate decision-making.

This study aims to clarify the information needed for surrogate decision-making and the support needs of healthcare professionals.

Additionally, this study was previously published on the Research Square preprint server on April 10, 2024 [14].

## Materials and methods

Study design and definition of the term

This cross-sectional study used a questionnaire. In this study, “surrogate decision-making” was defined as follows: a patient's family member or other person designated by the patient makes decisions about the course of treatment on behalf of the patient who is incapacitated. The content of the treatment is a decision that affects the patient’s life and prognosis. This includes information on life-saving procedures and withholding or interrupting treatment.

Participants

This study used an online panel recruited through a private Japanese research company. The inclusion criteria for the participants were as follows: (1) participants between 18 and 79 years of age who had experience in making decisions on behalf of patients during hospitalization and (2) patients with malignant tumors, neurological disorders, and neurosurgical diseases, cardiovascular medical and other surgical diseases, and emergency disease. The exclusion criteria were as follows: (1) the participant had a history of being diagnosed with cognitive problems, and (2) the participant was currently unable to lead an independent life.

Sample size

This survey aimed to collect information from family members with experience in making surrogate decisions. Information about how they made such decisions and what information they required to do so was collected. Previous studies revealed that families of seriously ill patients tended to insist more strongly on their own wishes than on those of their patients [4,5]. For this reason, this study aimed to evaluate the correlation between the weight of the wishes of the family and those of the patient in surrogate decision-making. Assuming a power of 0.8, a significance level of 0.05, and r = 0.4 [15], the required sample size was calculated to be 51.

Recruitment methods and study procedures

**Figure 1:**
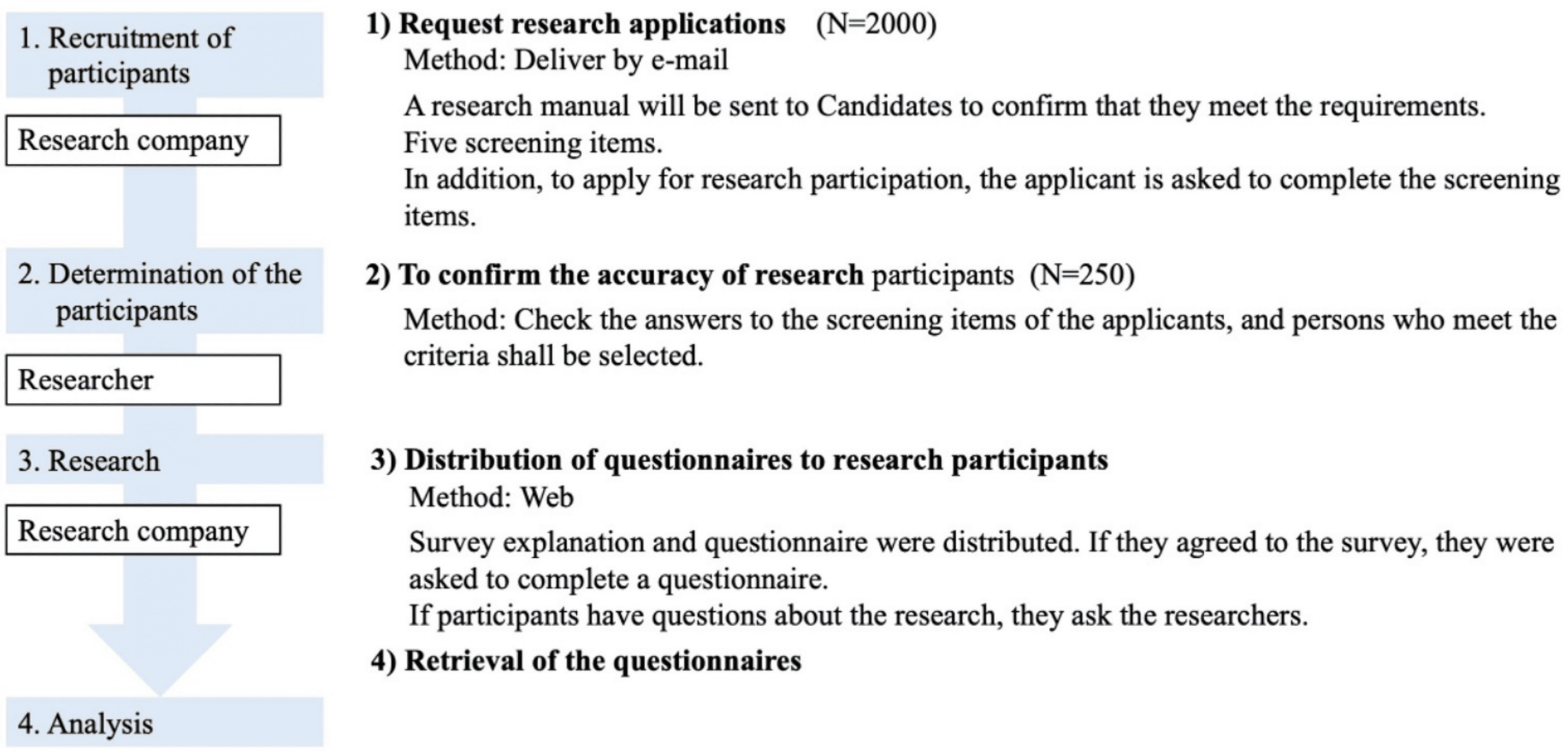
Study process.

The procedure used in this study is illustrated in Figure 1. Candidates were recruited online through a research company with a panel of “Family members of patients who are visiting a doctor or staying in hospital due to any illness.” At the time, the population was 2,000 people. Candidates were asked to read an online document outlining the research before applying for the study. Next, the candidates answered five screening questions. The screening items were: (1) whether you have experienced surrogate decision-making for hospitalized patients; (2) the name of the patient’s disease; (3) the content of the surrogate decision-making; (4) whether you have experienced a diagnosis such as cognitive decline or dementia; and (5) whether you are currently able to lead an independent life. Additionally, the researcher checked whether the screening items they answered met the criteria. A research company conducted simple random sampling from these candidates and selected 250 participants. This number was determined based on the expectation that the recovery rate would be approximately 50%. The research explanation and questionnaire were then presented to participants online. If the participants agreed to participate, they completed an online questionnaire. The responses were closed when a sample size of 60 was obtained. Data collection was carried out from February to March 2023.

Survey items

The study surveyed (1) details of when the surrogates made decisions and (2) perceptions of life-sustaining treatment and resuscitation measures. The survey items were answered using descriptive formulas regarding the content of surrogate decision-making and the patient’s disease. In addition, decision-making tools were selected from these options. Moreover, the support they wanted to receive from healthcare professionals was determined using an open-ended questionnaire. Regarding the quality of decision-making, the degree of satisfaction and stress in surrogate decision-making were assessed using a numeric rating scale ranging from 0 to 10. Furthermore, respondents answered questions about their experiences discussing life-sustaining treatments with patients, knowledge of life-sustaining treatments and resuscitation measures, and confidence in making surrogate decisions. Using the Control Preferences Scale as a basis [16], I created options and investigated surrogates’ preferences for decision-making roles. The characteristics of the participants included sex, age, employment status, education, and family status.

Data analysis

For the analysis of the questionnaire, basic statistics were calculated using SPSS version 25 (IBM Corp., Armonk, NY) for numerical data. After basic statistics were calculated, normality was confirmed, and descriptive statistics were obtained. Descriptive statistics were paired with t-tests and χ2 tests for nominal scales. Significance was set at <5% (two-tailed).

The free-text data were subjected to content analysis using NVivo (Lumivero, Denver, CO), and the prevalence of descriptions was confirmed. The content analysis used in this study is characterized by objectively, systematically, and quantitatively describing content [15]. In the process of analyzing the research results, the author received advice from members of the research team to which he belongs and from experts in qualitative research to improve the accuracy of the results.

Ethical considerations

The study was approved by the Ethics Committee at St. Luke’s International University (approval number: 22-A101) and conducted following the Declaration of Helsinki guidelines. Participants were informed in writing about the purpose of the study, research methods, participation in and withdrawal from the study, personal information protection and data management, publication of results, and withdrawal of consent. Basic attribute data were coded and kept anonymous so that the data could not be identified.

## Results

Sixty participants were included in the analysis (Table 1). The mean age (standard deviation) of the participants was 55.3 ± 13.0 years, and 42 (70.0%) were men. As for the preference regarding decision-making roles, “Me and the physician decide together equally” was the most common at 26 (43.3%).

**Table 1:** Characteristics of the study participants.

Variables	Total (N = 60)	
	n	%
Region of Japan		
Hokkaido	3	5.0
Tohoku	5	8.3
Kanto	16	26.7
Chubu	10	16.7
Kinki	18	30.0
Shikoku	4	6.7
Chugoku	1	1.7
Kyusyu	2	3.3
Age of categories		
20-29	3	5.0
30-39	6	10.0
40-49	7	11.7
50-59	17	28.3
60-69	18	30.0
70-79	9	15.0
Age (mean ± standard deviation)	55.3 ± 13.0	
Gender		
Men	42	70.0
Women	18	30.0
Occupation		
Full-time	36	60.0
Part-time	8	13.3
Unemployed	16	26.7
Highest level of education		
High school	14	23.3
Two-year college	5	8.3
Career college	7	11.7
College/university	31	51.7
Graduate	3	5.0
Family		
Have family living together and have family separated	42	70.0
Have family members living together but not separated	9	15.0
No family living together but have separated family	7	11.7
No one	2	3.3
Preference of decision-making		
Physicians decide for themselves based on their own knowledge	2	3.3
The physician decides, but takes my opinion into account	15	25.0
The physician and I decide together on an equal basis	26	43.3
I decide, taking into account the physician's opinion	16	26.7
I decide on my own, based on my own knowledge	1	1.7

Current status of surrogate decision-making by family members

Family members were asked to select one case involving surrogate decision-making (Table 2). When family members made surrogate decisions, 15 patients (25.0%) had cranial nervous system diseases. Regarding the content of decision-making, 27 (45.0%) concerned life-sustaining treatment, and 10 (16.7%) concerned emergency surgery. There was a significant difference in the sense of satisfaction with surrogate decision-making between decisions in line with the patient's wishes (7.7 ± 2.7) and the family's wishes (6.6 ± 2.8) (P = 0.005). A positive correlation was found between these variables (r = 0.349, P < 0.01) (Table 3). Furthermore, the degree of emotional burden experienced when families made surrogate decisions was 7.9 ± 2.7. Furthermore, there was a negative relationship between the family's understanding of the patient's treatment and the mental stress related to decision-making.

**Table 2:** Current situation when family members make surrogate decisions. ^*1^: Paired t-test (p-values are significant at the level of ≤ 0.05). ^*2^: The answers were rated on a scale of 0-10. ^*3^: Duplicate answers.

Variables	Total (N = 60)			P-value^*1^
	n	%		
Disease of the patient				
Cardiovascular	7	11.7	-	-
Neurology and neurosurgery	15	25.0	-	-
Gastroenterology	10	16.7	-	-
Kidney and urinary	6	10.0	-	-
Respiratory	8	13.3	-	-
Hematology	2	3.3	-	-
Infection	2	3.3	-	-
Trauma	10	28.3	-	-
Psychiatric dementia	9	15.0	-	-
Malignant disorder	17	28.3	-	-
Contents of surrogate decision-making				
Life-sustaining treatment	27	45.0	-	-
Emergency surgery	10	16.7	-	-
Attached ventilator	5	8.3	-	-
Brain death determination and organ transplantation	2	3.3	-	-
Withholding or withdrawing				
Nutrition such as gastrostomy	7	11.7	-	-
Artificial dialysis	1	1.7	-	-
Life-supporting device	2	3.3	-	-
Others	6	10.0	-	-
The decision was made according to the family's wishes^*2^ (Mean ± standard deviation)	6.6 ± 2.8		t = -2.91	0.005
The decision was made according to the patient's wishes ^*2^ (Mean ± standard deviation)	7.7 ± 2.7			
The degree of mental burden when making surrogate decisions ^*2^ (Mean ± standard deviation)	7.9 ± 2.7		-	-
What was referred to when making surrogate decisions ^*3^				
Discuss among family members	49	81.7	-	-
Talk counsel with friend or someone trust	10	16.7	-	-
Refer to information in books, press, etc.	3	5.0	-	-
Refer to information on the internet	13	21.7	-	-
Refer to information on television	0	0.0	-	-
Referring to information from newspapers	1	1.7	-	-
Referring to information discussed with the patient	18	30.0	-	-
Information from healthcare professionals	5	8.3	-	-
Information from family who have had the same experience	1	1.7	-	-
I don't have anything	3	5.0	-	-
Which healthcare professional have you consulted? ^*3^				
Primary physician	41	68.3	-	-
Physician other than the primary physician	7	11.7	-	-
Nurse (in-hospital nurse)	8	13.3	-	-
Nurse (home care nurse)	3	5.0	-	-
Social worker	6	10.0	-	-
Pharmacist	2	3.3	-	-
Physical therapist	1	1.7	-	-
Care manager	1	1.7	-	-
I have not consulted	10	16.7	-	-

**Table 3:** Perceptions and item correlations for surrogate decision-making. ACP, advance care planning; ** P < 0.01; * P < 0.05. N = 60. Spearman's rank correlation coefficient. *7: Number of treatments that are recognized as being understood (max = 8, min = 0, average = 6.2).

	*1	*2	*3	*4	*5	*6
*1. The decision was made according to the family's wishes	-	-	-	-	-	-
*2. The decision was made according to the patient's wishes	0.349**	-	-	-	-	-
*3. The degree of mental burden when making surrogate decisions	-0.015	0.113	-	-	-	-
*4. Participant's age	0.128	0.105	0.180	-	-	-
*5. It's hard to discuss ACP with patients	0.128	0.005	0.130	0.109	-	-
*6. I want to have the opportunity to discuss ACP with patients	0.235	0.268*	0.185	0.029	-0.090	-
*7. Number of treatments that are recognized as being understood	-0.101	0.042	-0.394**	-0.080	-0.348**	-0.136

Moreover, discussions among family members were the most common reference points for making surrogate decisions, with 49 cases (81.7%). In addition, I inquired about the support that family members wanted to receive when making surrogate decisions (Table 4). Nineteen (31.7%) answered that the support from healthcare professionals was sufficient. Highly needed support included prognostication, information on available treatments and options, and prospects for progress and recovery after each treatment.

**Table 4:** What support did you hope to receive from healthcare professionals when making a surrogate decision? Free opinions about the support they wanted from healthcare professionals and the rate of its term's occurrence. N = 60.

Variables			n	%
I had enough support from the healthcare professionals.			19	31.7
I have the support I hoped to receive from the healthcare professionals.			41	68.3
Healthcare professional		Prognostic prediction	22	53.7
		Explanation of the current situation/possible treatment	20	48.8
		Provision of appropriate information	20	48.8
		Prognosis and recovery prospects by treatment	18	43.9
		Multiple options of treatment	11	26.8
		Proposal for palliative care	9	22.0
		Second opinion	5	12.2
		Decisions made by healthcare professionals	4	9.8
		I need time to think.	3	7.3
		Presentation of similar cases and their results.	3	7.3
		I need you to be close to me.	3	7.3
		I want you to consult with me.	2	4.9
		The possibility of sudden change worsens, and the reason	2	4.9
Family		Mental support	10	24.1
		How to make a decision	8	19.5
		The unification of family member's wishes	6	14.6
		Medical cost	3	7.3
Patient		Probability and degree of recovery	15	36.6
		Will patients recover until they can return to their daily activities?	12	29.3
		The condition of a patient not feeling pain	11	26.8
		Being able to receive appropriate treatment	7	17.1

Perceptions of life-sustaining and resuscitative treatments

Twenty-six (43.3%) family members did not understand the assisted circulation devices (Table 5). Regarding respiratory care, 53 (88.3%) participants answered that they did not understand noninvasive positive pressure management. The most common procedure recognized as being included in “cardiopulmonary resuscitation” was cardiac massage by 50 respondents (83.3%). In addition, the difficulty of asking patients about the medical care they would like to receive in a life crisis differed among participants. Additionally, regarding the perception of important information and values when making surrogate decisions, 42 (70.0%) surrogates most often mentioned whether the patient’s consciousness would recover with treatment. The patient’s age was 40 (66.7%), the survival rate was 30 (50%), and the age of the family was 28 (46.7%).

**Table 5:** Perceptions of surrogate decision-makers on the patient's emergency care. ACP: advance care planning. ^*1^: Duplicate answer items. ^*2^: The answers were rated on a scale of 0-10. ^*3^: Other, the condition of a patient not suffering.

Variables	Total (N = 60)	
	n	%
Treatment that could not be well-understood.^*1^		
Arrival resuscitation	9	15.0
Mechanical ventilation	9	15.0
Assist circulation device	26	43.3
Dialysis	9	15.0
Drugs to maintain blood pressure	10	16.7
Nutrition (tube feeding, etc.)	11	18.3
Blood transfusion and Infusions	7	11.7
Infusions	22	36.7
Nothing	19	31.7
Respiratory treatment that could not be well-understood.^*1^		
Intubate trachea	12	20.0
Have a trach	14	23.3
Non-invasive positive airway pressure ventilation	53	88.3
Procedures recognized as part of "cardiopulmonary resuscitation."^*1^		
Artificial breath	45	75.0
Intubate trachea	40	66.7
Mechanical ventilation	45	75.0
Chest compression	50	83.3
Automated external defibrillator or defibrillator	47	78.3
Drugs to maintain blood pressure	25	41.7
Nutrition (tube feeding, etc.)	7	11.7
Blood transfusion	9	15.0
Infusions	7	11.7
Nothing	3	5.0
I want to discuss advanced care planning with my family.^*2^ (Mean ± standard deviation)	8.3 ± 2.4	
Who do you consult when making surrogate decisions?^*1^		
Marital partner/partner	37	61.7
Children	21	35.0
Brother/sister	25	41.7
Own parents	15	25.0
Partner's parents	6	10.0
Relatives	7	11.7
Friends	10	16.7
Others	2	3.3
No one	1	1.7
How difficult is it for you to discuss ACP with patients?^*2^ (Mean ± standard deviation)	5.8 ± 3.0	
Information and values that are essential when making surrogate decisions.^*1^		
My age	28	46.7
Patient's age	40	66.7
Medical cost	16	26.7
Recovery of consciousness	42	70.0
Patient survival rate	30	50.0
Amount of patient care performed by family members	19	31.7
Nothing	4	6.7
Other^*3^	3	5.0

## Discussion

This study revealed the understanding of information, perception of values, and the quality of decision-making in the process of family members making surrogate decisions. Several previous studies have indicated that surrogates may make treatment decisions that are not in line with the patient’s wishes [17]. However, in this study, families who had experienced surrogate decision-making perceived that they made decisions that prioritized the patient’s wishes. There are two reasons for this observation. First, the decision support for the family by healthcare professionals may have been appropriately helpful. Second, many participants had experience in surrogate decision-making regarding life-sustaining treatments. Many families might have had more time to decide and consider the patient’s wishes. The most common reference for surrogate decision-making is discussion among family members. One conjecture is that decision-making was not based on medical information alone. Family members who make surrogate decisions regarding patient treatment bear a huge responsibility and burden [18]. Considering the patient’s autonomy by discussing the topic among the family members is essential [19]. In addition to helping families work together to determine the best treatment for patients, tools to share the process with healthcare professionals could improve the quality of surrogate decision-making. Moreover, learning programs and education on how to support families of end-of-life patients are being developed [20], and decision-making support from healthcare professionals and psychological support for families may have been provided.

It is important to educate surrogates on how to make decisions that respect the patient’s autonomy [18]. It is essential to support the family in making the best treatment decision for the patient while also listening to their wishes and thoughts and reinforcing their mental care so that the decision is acceptable to the patient and the family [21]. This study found that when there is a lack of understanding of the treatment, there is a tendency for the family to experience greater emotional distress after the decision-making process. The data to support this finding in detail are not clear in this study. However, to prevent the family from feeling more regret after making a decision, it may be necessary for the medical staff to make an effort to check and enhance the family's understanding of the treatment.

It is also evident that even if family members recognize that life-sustaining treatment is not appropriate for the patient, they may find it difficult to make life-saving or life-sustaining surrogate decisions regarding the loss of their loved one [22,23]. Healthcare professionals should be encouraged to simultaneously provide care related to family member crises and anticipatory grief during the surrogate decision-making process. In addition, patients and their families often do not have prior experience with the ACP process.

Therefore, the challenge is that the surrogate can only speculate about the patient's intentions [24]. I believe that coordinating with the patient and family to discuss ACP early in the treatment initiation phase represents necessary support by healthcare professionals. Furthermore, surrogate decision-makers might make decisions with an inadequate understanding of life-saving treatments. When a patient's condition suddenly changes, there is often insufficient time to make treatment decisions [25]. Surrogates are expected to make critical decisions while dealing with crises and anxiety caused by the event. Healthcare professionals need to assist the family in making surrogate decisions that respect the patient’s autonomy while providing emotional support for the surrogate’s crisis state [18,22]. Healthcare professionals and family members often do not have the same perceptions regarding treatment [26]. Providing appropriate and understandable information and explanations, even under time constraints, is essential for healthcare providers [27]. This process has been shown to enhance shared decision-making [28]. However, providing decision-makers with the necessary information and understanding under time constraints is difficult. It may be useful to utilize decision aids that supplement healthcare professionals’ explanations and assist in complex decision support. In this case, the key is for healthcare professionals to understand the values held by the patient and the surrogate [28]. In this study, the surrogates identified important information and values when making decisions. It might be useful for healthcare professionals to include this information when facilitating shared decision-making and to present the benefits and risks associated with treatment options to the surrogate decision-maker.

Recently, abundant information has become available, such as via the internet and social networking services, making it more difficult to gather correct information [29]. Healthcare providers are important in ensuring that evidence-based information is accurately communicated to patients and surrogate decision-makers [30]. In this regard, it is necessary to know that the information is not simply about the treatment but also the patient's benefits and risks. Furthermore, it is necessary to use tools such as pamphlets with visual aids to facilitate understanding of the information [29]. In addition to circulating these tools, I believe that nurses and other healthcare professionals should support them by providing counseling and coaching, thereby supporting surrogate decision-making that aligns with patients’ values.

Limitations

Although I endeavored to collect cross-sectional information from Japan, the sample size was small. In addition, the details of the medical support received when surrogate decision-making was performed were unclear. Therefore, it was impossible to determine the level of effective medical support for the surrogate decision-makers. Furthermore, I believe that when making a surrogate decision, it is necessary to be fully informed and understand the benefits and risks of the options; however, this point could not be clarified and may be a factor that biased the results.

## Conclusions

The findings of this study suggest that supporting families in making proxy decisions has the potential to increase the satisfaction of family members by encouraging decision-making that puts the patient's wishes first. Additionally, encouraging families to understand the patient's medical information may reduce mental stress after the decision has been made.

For surrogate decision-making, not only involvement with healthcare professionals but also discussion among family members are important factors, suggesting that it may be necessary to build a support system, including facilitating agreement among family members’ thoughts and perceptions, for the provision of patient-centered care.
